# Prevalence, antibiogram, and risk factors of methicillin-resistant *Staphylococcus aureus* (MRSA) asymptomatic carriage in Africa: a systematic review and meta-analysis

**DOI:** 10.1186/s12879-025-10819-4

**Published:** 2025-04-11

**Authors:** Ahmed Azzam, Heba Khaled, Heba Mohamed Fayed, Youssef Mansour, Mariam Eldalil, Eslam Elshennawy, Haitham Salem, Hoda A. Elkatan

**Affiliations:** 1 Department of Microbiology and Immunology, Faculty of Pharmacy, Helwan University, Cairo, Egypt; 2 Department of Biochemistry, Faculty of Pharmacy, Cairo University, Cairo, Egypt; 3 Department of Oral & Maxillofacial Surgery, Faculty of Dentistry, October 6 University, Cairo, Egypt; 4 Intern doctor, Mansoura University Hospitals, Mansoura, Egypt; 5 Department of Public Health & Community Medicine, Faculty of Medicine, Cairo University, Cairo, Egypt; 6 Department of Gastroenterology and Hepatology, Faculty of Medicine, Kafr El-Sheikh University, Kafr El-Sheikh, Egypt; 7 Faculty of Medicine, Ain Shams University, Cairo, Egypt; 8 Department of Pediatrics, Aboukir General Hospital, Alexandria, Egypt

**Keywords:** Methicillin-resistant *Staphylococcus aureus*, MRSA, Carriage, Colonization, Resistance, Meta-Analysis, Africa

## Abstract

**Background:**

MRSA represents a significant public health challenge, particularly in resource-constrained regions like Africa. A critical factor in its spread is the role of asymptomatic carriers, who not only facilitate transmission but also face a markedly higher risk of developing MRSA-related infections. Against this backdrop, the current meta-analysis provides a comprehensive evaluation of MRSA colonization rates, associated risk factors, and antibiotic resistance profiles across African populations.

**Methods:**

A comprehensive literature search was conducted across African Journals Online, African Index Medicus, PubMed, Scopus, Google Scholar, and Web of Science from January 1, 2014, to January 1, 2025. Eligible studies reported on MRSA colonization rates, associated risk factors, or antibiotic resistance patterns within African populations. Results were presented as pooled prevalence or risk ratios (RR) with 95% confidence intervals, employing a random-effects model in R software (meta package). A p-value of < 0.05 was considered statistically significant. The study followed the PRISMA guidelines throughout.

**Results:**

Sixty-nine studies with 23,484 participants from 16 African countries were included. Subgroup analyses identified Healthcare Workers and hospitalized patients as having the highest pooled prevalence at 13.6% and 12.9%, respectively. Conversely, lower prevalence rates were observed among healthy community residents and children, at 4.1% and 4.7%, respectively. Among HCWs, Egypt reported the highest MRSA colonization rate at 18.1%. Key risk factors for MRSA colonization include a history of hospitalization (RR: 2.2), prior antibiotic use (RR: 1.4), diabetes mellitus (RR: 4.4), HIV with CD4 < 200 cells/µL (RR: 2.8), invasive procedures (RR: 4.8), and being a nurse compared to a physician (RR: 1.8), all with *p* < 0.05. Antibiotic resistance of MRSA was low for linezolid (2.7%) and vancomycin (5.9%), but higher for mupirocin (10.7%), clindamycin (23.6%), and Trimethoprim/sulfamethoxazole (38.9%).

**Conclusion:**

MRSA colonization is a significant public health challenge in Africa, particularly among healthcare workers and hospitalized patients. Implementing targeted interventions for these high-risk groups can effectively reduce MRSA transmission and overall infection burden. Continuous monitoring is essential, especially given the resistance to mupirocin, a key antibiotic used for MRSA decolonization.

**Supplementary Information:**

The online version contains supplementary material available at 10.1186/s12879-025-10819-4.

## Introduction

Antimicrobial resistance (AMR) is a critical global public health issue, recognized as one of the top ten threats to human health worldwide [1, 2]. Among the most significant contributors to this crisis is methicillin-resistant *Staphylococcus aureus* (MRSA). According to the World Health Organization (WHO), individuals infected with MRSA face a 64% higher risk of mortality compared to those with non-resistant infections [1]. This elevated risk is mainly attributed to limited treatment options and delays in administering appropriate empirical antibiotic therapy. The gravity of MRSA is underscored by its classification as a ‘serious threat’ in the Centers for Disease Control and Prevention’s (CDC) 2019 Antibiotic Resistance Threat Report and its inclusion on the WHO’s 2024 high-priority pathogen list, emphasizing the urgent need for accelerated antibiotic development [3, 4]. This growing threat not only endangers individual lives but also places immense strain on healthcare systems globally.

*S. aureus* can asymptomatically colonize the human body, preferentially the nasal cavity, with colonized individuals serving as key reservoirs for transmission, particularly in healthcare settings where they may inadvertently disseminate the pathogen to vulnerable populations [5–8]. Evidence from previous studies indicates that colonization with either MSSA or MRSA represents a significant risk factor for subsequent infection [5–8].

Effective approaches to reducing the MRSA infection burden include decolonization of carriers through either universal decolonization (applied to all patients regardless of MRSA status) or targeted decolonization (limited to identified MRSA carriers through screening). The Society for Healthcare Epidemiology of America (SHEA) and the Infectious Diseases Society of America (IDSA) strongly recommend universal decolonization for all adult ICU patients, while targeted decolonization is advised for high-risk populations, such as patients undergoing implant surgeries [9]. Decolonization antibiotics like mupirocin nasal ointment and antiseptic body washes are often used to reduce MRSA carriage. However, their effectiveness varies, and resistance to mupirocin has been reported [10–12]. This underscores the importance of accurately quantifying the burden of MRSA carriers and assessing their resistance profiles against key treatment options to inform effective intervention strategies.

AMR presents a particularly formidable challenge in resource-limited settings such as Africa, where healthcare disparities, inadequate antibiotic stewardship programs (ASPs), insufficient infection control measures, and the widespread practice of antibiotic self-medication significantly exacerbate the problem [13–18]. To address the lack of comprehensive data and overcome the limited statistical power of individual studies, we conducted a meta-analysis. Our objectives were to quantify the prevalence of MRSA carriage across Africa, analyze the AMR profile of MRSA against key treatment options, and identify risk factors associated with MRSA colonization. The findings offer a detailed overview of the epidemiology of MRSA carriage on the continent, highlighting populations at higher risk where targeted interventions should be prioritized. Moreover, our analysis sheds light on MRSA resistance patterns, including resistance to decolonizing agents such as mupirocin. By filling critical knowledge gaps, this study provides actionable insights to inform policy development, enhance ASPs, and strengthen infection prevention and control strategies in Africa.

## Methods

### Search strategy

A comprehensive literature search was conducted using the following databases: African Journals Online, African Index Medicus, PubMed, Scopus, Google Scholar, and Web of Science. The search covered the period from January 1, 2014, to January 1, 2025, to include up-to-date data and to reflect current trends in MRSA carrier rate. The detailed search strategy is presented in Table S1. It was adapted to align with the specific requirements and functionalities of each database. An example of the detailed search strategy for the Scopus and PubMed databases is provided in Table S2. This study followed the PRISMA (Preferred Reporting Items for Systematic Reviews and Meta-Analyses) guidelines [19]. Table S3 present the PRISMA Main Checklist (27-item checklist).

### Eligibility criteria

#### (a) inclusion criteria

Studies were included if they met the following criteria: (1) provided data on MRSA colonization rates, associated risk factors, or MRSA antibiograms; (2) were conducted in African countries; and (3) utilized valid methods for MRSA detection.

#### (b) exclusion criteria

Studies were excluded if they met any of the following criteria: (1) focusing exclusively on MRSA infections, (2) involving specimens from food or animals, or (3) being literature reviews, preprints, or conference abstracts.

Two authors (A.A. and H.K.) selected the included articles based on the previously mentioned eligibility criteria, which were then cross-checked by another group of two authors (H.M.F. and Y.M.).

### Data extraction

Data extraction was independently performed by two investigators (M.E. and E.E.) and subsequently cross-verified by two additional reviewers (H.S. and H.A.E.) to ensure accuracy.

For each included study, the following details were collected: the first author’s last name, study period, country, type of population, number of screened participants, participants’ age categories, total number of MRSA cases, specimen type, and risk factors associated with MRSA colonization. Additionally, the antibiogram of MRSA isolates was recorded for the following antibiotics, which are commonly used in managing MRSA infections: linezolid, vancomycin, ceftaroline, telavancin, clindamycin, trimethoprim-sulfamethoxazole (TMP-SMX), rifampin, tetracycline, dalbavancin, oritavancin, daptomycin, tedizolid, fusidic acid, and mupirocin [20].

### Quality assessment

The quality of the included studies was meticulously assessed by three reviewers (H.M.F., Y.M., and M.E.) using the Joanna Briggs Critical Appraisal Checklist for Prevalence Studies [21]. This assessment was later verified by three additional reviewers (E.E., H.S., and H.A.E.). The original checklist items are detailed in Table S4. This checklist systematically evaluated key aspects, including the appropriateness of the sampling frame, sampling methods, and sample size; the description of study settings; the adequacy of data analysis coverage; the use of validated methods for MRSA identification; the standardization of MRSA detection methods; and the adequacy of the response rate. We considered a cutoff score of 6 out of 9 as the threshold for a study to be classified as fair quality.

### Data synthesis

A random-effects model employing the inverse variance weighting technique was utilized to calculate pooled prevalence rates and 95% confidence intervals (CIs) or risk ratios, depending on the type of data analyzed. Only studies with at least three prevalence estimates were included in the meta-analysis to improve generalizability and reliability. The random-effects model was chosen to account for heterogeneity among studies arising from variations in populations, settings, and methodologies. Predefined subgroup analyses were performed according to the type of population. Additionally, the resistance profiles of MRSA isolates were evaluated for the following antibiotics: linezolid, vancomycin, ceftaroline, telavancin, clindamycin, TPM-SMX, rifampin, tetracycline, dalbavancin, oritavancin, daptomycin, tedizolid, fusidic acid, and mupirocin. Heterogeneity across studies was assessed using the I-squared (I²) statistic, with I² values exceeding 75% interpreted as indicative of substantial heterogeneity. To evaluate the robustness of the results, sensitivity analyses were performed using a leave-one-out approach. All statistical analyses were conducted using R software (version 4.4.1). A p-value below 0.05 was considered statistically significant.

## Results

### Characteristics of included studies

**Fig. 1:**
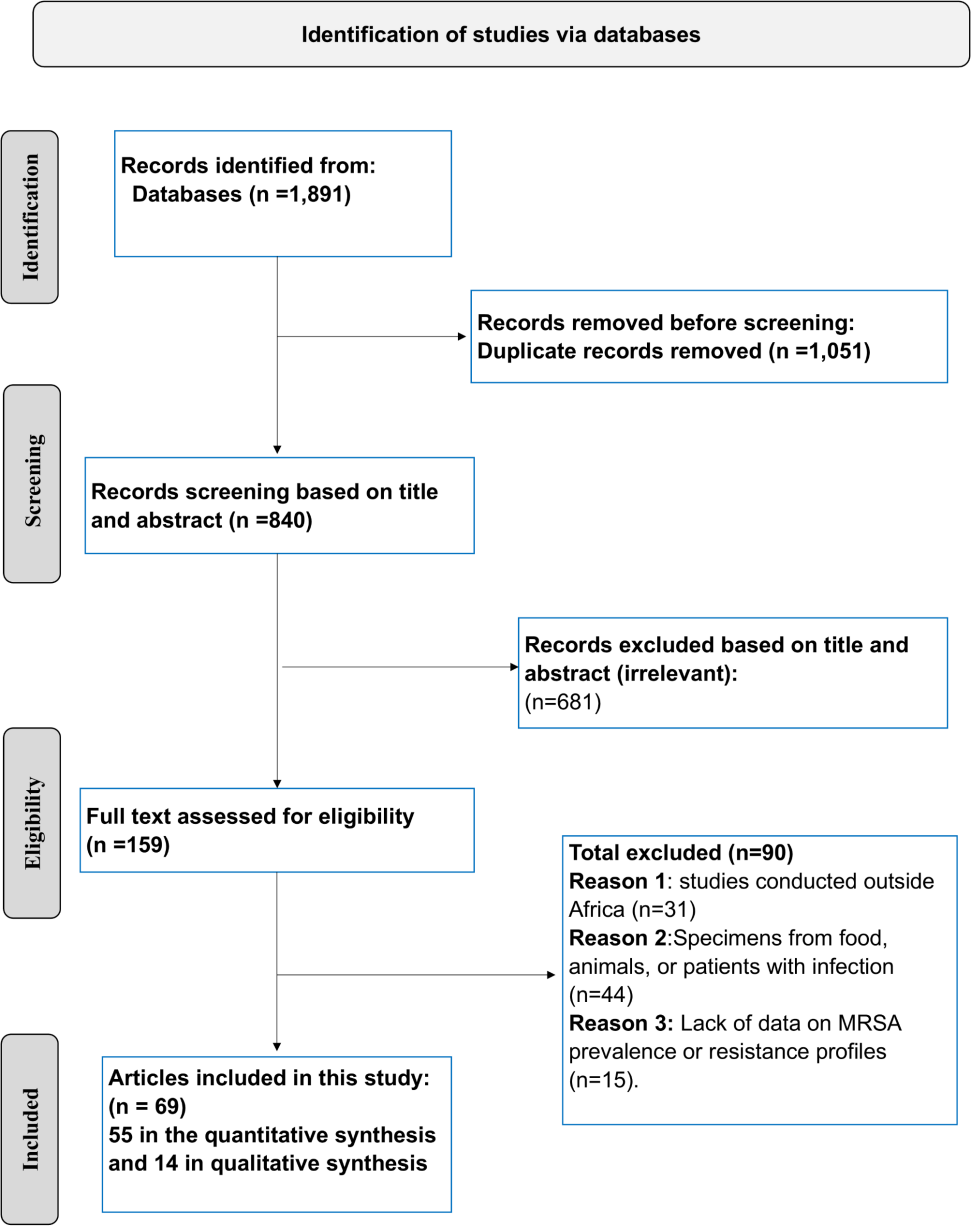
PRISMA flow diagram illustrating the selection process for the included articles

Out of 1,891 articles screened, 69 studies from 16 African countries met the eligibility criteria and were included in this meta-analysis, as shown in Fig. 1. The characteristics of the included studies and the quality assessment are presented in Table S5. The analysis comprised a total of 23,484 participants, among whom 1,766 were identified as MRSA carriers. The studies were distributed as follows: 15 from Egypt [22–36], 16 from Nigeria [37–52], 12 from Ethiopia [53–64], 7 from Libya [65–71], 3 from Tanzania [72–74], 2 from Morocco [75, 76], 2 from Algeria [77, 78], 2 from Kenya [79, 80], and2 from Uganda [81, 82]. Additionally, there was one study each from Sudan [83], Ghana [84], Madagascar [85], South Africa [86], the Republic of Congo [87], Namibia [88], Angola [89], and Botswana [90].

Among the included groups, healthcare workers (HCWs) were represented by the highest number of estimates (*n* = 23) [22, 24, 26–33, 37, 38, 49, 54, 63, 67–69, 71, 74, 82, 83, 85], followed by children (*n* = 13) [42, 50, 53, 58–60, 64, 69, 72, 75, 84, 88, 89], university students (*n* = 13). Among university students, 5 studies focused on medical or agricultural students with significant exposure to hospitals or animal farms [34, 44, 51, 55, 56], 4 studies on non-medical or agricultural students [33, 44, 46, 83] and 4 studies on university students with no specified data [41, 43, 47, 79]. Other groups include hospitalized adult patients (*n* = 8) [27, 32, 33, 36, 66, 78, 80, 86], healthy community residents (*n* = 6) [27, 46, 77, 80, 83, 87], HIV-positive patients (*n* = 4) [40, 61, 81, 90], and hospital janitors (*n* = 3) [32, 57, 62].

Additionally, two studies focused on hemodialysis patients [24, 76], non-hospital janitors [57, 62], and outpatients [23, 65]. One study was reported for each of the following groups: renal transplant recipients [70], elderly individuals in hospital and nursing home settings [39], atopic dermatitis patients [25], emergency department patients [73], and a combined group of university students and healthcare workers without satisfaction data [52]. Regarding the quality assessment, all included studies scored 6 or higher out of 9, which we considered indicative of fair quality. The studies with lower scores typically lost points due to smaller sample sizes and unclear response rates, as shown in Table S5.

### Subgroup analysis of MRSA colonization across different populations in Africa

Subgroup analysis indicated notable differences in MRSA colonization prevalence among various populations, as shown in Table 1. HCWs exhibited the highest prevalence at 13.6% (95% CI: 9.4–18.4), with Egypt reporting the highest rate at 18.1% (95% CI: 12.7–24.3, 10 studies). This was followed by hospitalized adult patients, who showed a prevalence of 12.9% (95% CI: 5.4–22.9). Medical or agricultural students with significant animal farm or hospital exposure also showed a notable prevalence at 8.8% (95% CI: 3.5–16.1), as did hospital janitors at 8.4% (95% CI: 5.6–11.7).

Conversely, lower prevalence rates were found among other populations. HIV-positive individuals exhibited a prevalence of 5.1% (95% CI: 1.2–11.4). Children had a prevalence rate of 4.7% (95% CI: 2.3–7.8), while healthy community members recorded the lowest prevalence at 4.1% (95% CI: 1.7–7.4). Additionally, non-medical and agricultural students showed a prevalence of 4.6% (95% CI: 1.4–10.3).

**Table 1:** Meta-analysis of MRSA colonization across different populations

Subgroup	No. of studies/ Estimates	Proportion (%) 95%-CI (%)	I² (%)
**Healthcare workers**	**23** [22, 24, 26–33, 37, 38, 49, 54, 63, 67–69, 71, 74, 82, 83, 85]	13.6 [9.4; 18.4]	95.6
**Hospitalized Adult patients**	**8** [27, 32, 33, 36, 66, 78, 80, 86]	12.9 [5.4; 22.9]	97.5
Medical or Agricultural students*	**5** [34, 44, 51, 55, 56]	8.8 [3.5; 16.1]	91.4
**Hospital janitors**	**3** [32, 57, 62]	8.4 [5.6; 11.7]	0
**HIV-positive patients**	**4** [40, 61, 81, 90]	5.1 [1.2; 11.4]	92.7
**Children (**< 18 years)	**13** [42, 50, 53, 58–60, 64, 69, 72, 75, 84, 88, 89]	4.7 [2.3; 7.8]	95.0
**Non-Medical or Agricultural students**	**4** [35, 46, 48, 85]	4.6 [1.4; 10.3]	93.0
**Healthy community residents**	**6** [27, 46, 77, 80, 83, 87]	4.1 [1.7; 7.4]	91.8

* These students were extensively exposed to hospitals or animal farms during their training programs

### Findings from studies excluded from quantitative analysis

Several estimates were excluded from the meta-analysis due to the limited number of studies available; however, these estimates still offer valuable insights into MRSA colonization rates across diverse populations and highlight areas for further research. Among hemodialysis patients, one study from Egypt reported a colonization rate of 16.4%, while another from Morocco reported 1.4% [24, 76]. Non-hospital janitors in Ethiopia showed colonization rates of 1.4% and 5% in two separate studies [57, 62].For outpatients, a study from Egypt reported a colonization rate of 32%, compared to 1.9% reported in a study from Libya [23, 65]. Other specific groups also reported single estimates, including renal transplant recipients in Libya (33.6%) [70], elderly individuals in hospital and nursing home settings in Nigeria (4.3%) [39], atopic dermatitis patients in Egypt (46.6%) [25], emergency department patients in Tanzania (8.5%) [73], and a combined group of university students and healthcare workers in Nigeria without satisfaction data (2.7%) [52]. In addition, four studies on university students—three conducted in Nigeria and one in Kenya—reported MRSA colonization rates ranging from 11 to 22.6% [41, 43, 47, 79]. However, these studies did not provide data on the students’ MRSA colonization risk factors or clarify whether the students were from medical or agricultural disciplines.

### Meta-analysis of antimicrobial resistance of MRSA

The meta-analysis of MRSA resistance profiles revealed varying levels of resistance to different antibiotics. Linezolid showed the lowest resistance at 2.7%, followed by vancomycin at 5.9%. Mupirocin resistance was reported at 10.7%, while clindamycin exhibited a resistance proportion of 23.6%. Ciprofloxacin resistance was 38.6%, closely followed by TMP-SMX at 38.9% and tetracycline at 40.2%. Gentamicin resistance was slightly higher at 44.6%, while rifampin and erythromycin exhibited the highest resistance proportions at 62.7% and 67.2%, respectively. The pooled resistance rate, along with the number of MRSA isolates tested, is presented in Table 2.

**Table 2:** Meta-analysis of the antibiogram of methicillin-resistant *Staphylococcus aureus* (MRSA) resistance rates in asymptomatic carriers across African countries

Antibiotic	Included studies	Total tested MRSA Isolates	Proportion (%) 95%-CI (%)	I² (%)
**Vancomycin**	5 [22, 30, 31, 33, 38]	154	5.9 [0.2; 16.6]	75.6
**Linezolid**	6 [22, 31, 33, 38, 73, 74]	211	2.7 [0.3; 6.7]	33.6
**Clindamycin**	10 [22, 26, 30, 31, 33, 38, 53, 56, 74, 85]	313	23.6 [11.9; 37.7]	85.5
**TMP-SMX**	9 [22, 33, 38, 53, 56, 72, 74, 85, 89]	251	38.9 [25; 53.8]	80.3
**Tetracycline**	6 [22, 29, 33, 56, 72, 89]	141	40.2 [21.5; 60.5]	81.3
**Mupirocin**	6 [22, 26, 30, 39, 73, 74]	184	10.7 [4.4; 18.9]	50.7
**Rifampin**	4 [29, 31, 33, 89]	110	62.7 [24.9; 93.6]	93.6
**Ciprofloxacin**	8 [22, 26, 30, 31, 38, 72–74]	243	38.6 [21.4; 59.2]	86.3
**Erythromycin**	7 [22, 33, 38, 39, 56, 74, 84]	212	67.2 [43.5; 84.5]	78.3
**Gentamicin**	10 [22, 29, 30, 33, 38, 39, 56, 72–74]	273	44.6 [27.5; 63]	82.4

### Factors associated with MRSA colonization in Africa

**Fig. 2:**
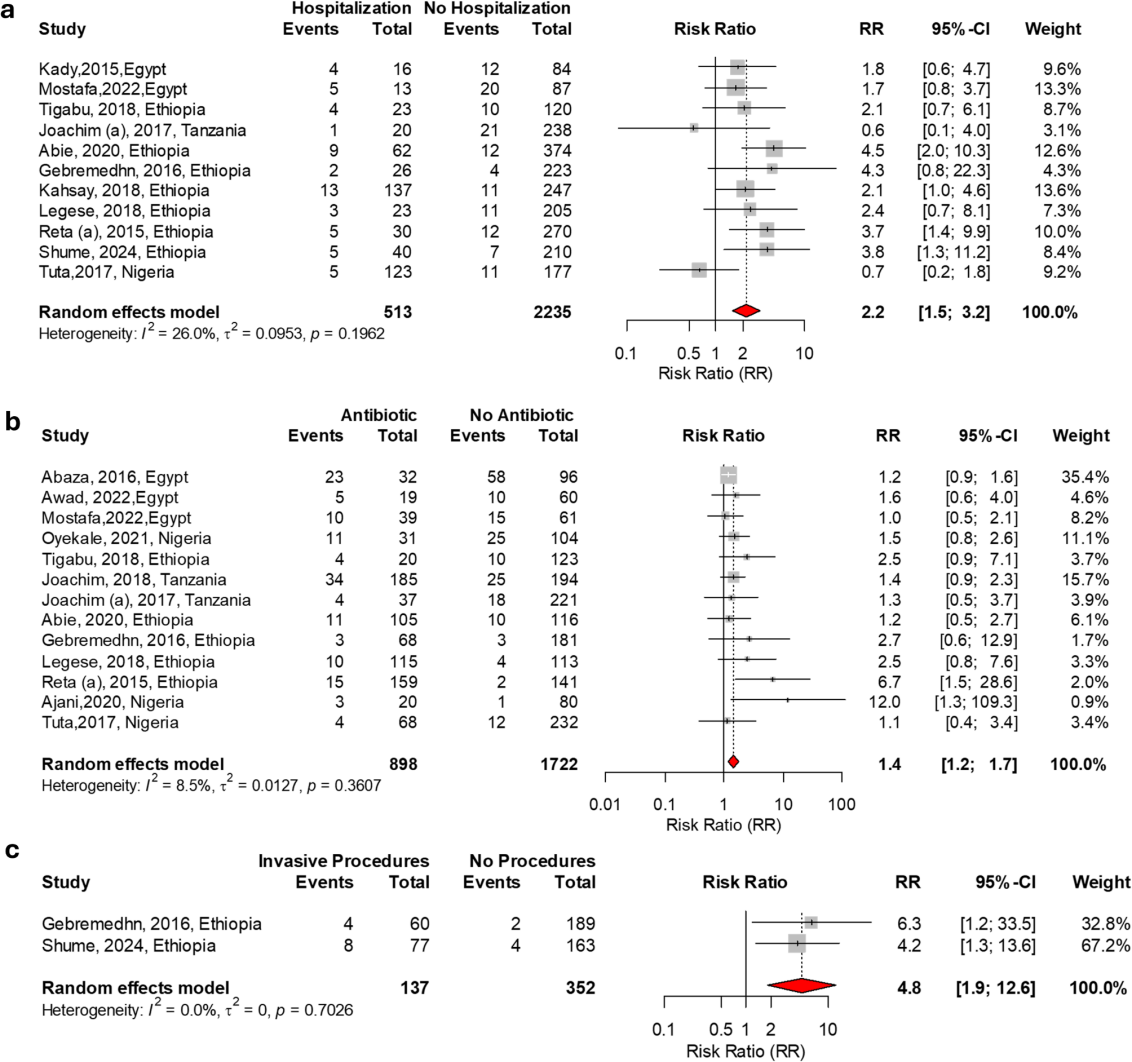
Significant factors associated with MRSA carriage in Africa. (**a**) Prior hospitalization vs. no hospitalization, (**b**) Prior antibiotic use vs. no antibiotic use, (**c**) Invasive procedures vs. no invasive procedures

**Fig. 3:**
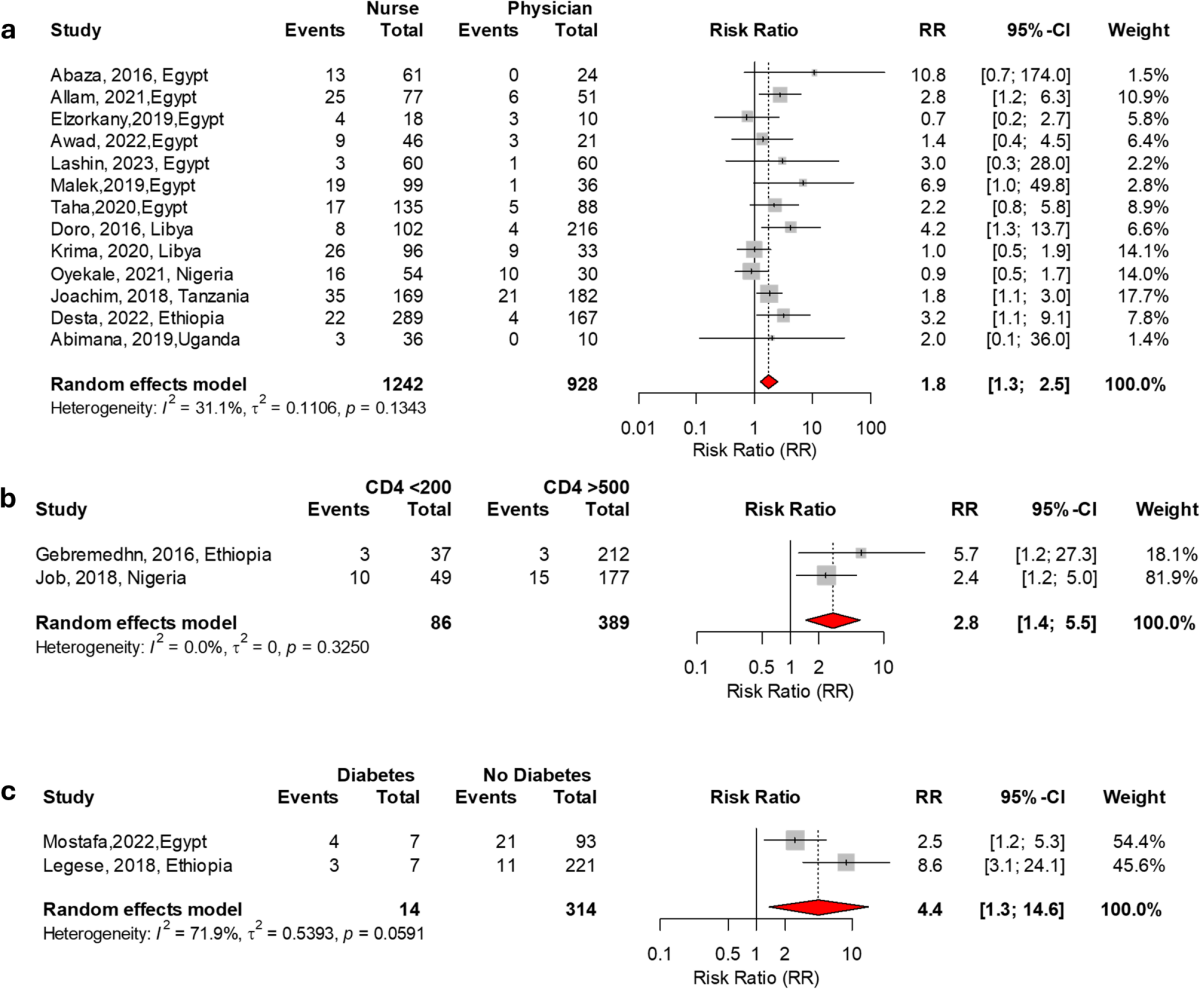
Significant factors associated with MRSA carriage in Africa: (**a**) Nurse vs. Physician, (**b**) HIV with CD4 < 200 cells/µL vs. > 500 cells/µL, (**c**) Patients with diabetes vs. no diabetes

The meta-analysis identified several significant risk factors for MRSA colonization in Africa, as shown in Table 3. Factors significantly associated with MRSA colonization included a history of hospitalization (RR = 2.2, *P* < 0.001), prior antibiotic use (RR = 1.4, *P* = 0.001), diabetes mellitus (RR = 4.4, *P* = 0.015), and HIV-positive status with a CD4 count below 200 cells/µL (RR = 2.8, *P* = 0.002). Invasive procedures were another notable risk factor (RR = 4.8, *P* = 0.001). Additionally, nurses had a higher risk of colonization compared to physicians (RR = 1.8, *P* = 0.005). The significant factors associated with MRSA carriage in Africa are presented in Figs. 2 and 3.

On the other hand, some risk factors were not statistically significant (*P* > 0.05). These factors included sex, where females had a non-significant risk compared to males (RR = 1.2, *P* = 0.11). Nasal allergy was associated with an increased risk (RR = 2.0, *P* = 0.15); however, this result lacked statistical significance. Smoking was not identified as a significant risk factor for MRSA colonization (RR = 0.9, *P* = 0.6). The sensitivity analyses confirmed that all pooled risk ratio estimates were robust, with no influential outliers identified.

**Table 3:** Meta-analysis of factors associated with methicillin-resistant *Staphylococcus aureus* (MRSA) colonization in African countries

Factor	Included studies	Pooled Risk ratio 95%-CI (%)	*P*-value	I² (%)
**a) Significant factors**				
History of hospitalization vs. No hospitalization	**11** [30, 34, 42, 53, 55, 57, 58, 61–63, 73]	2.2 (1.5 − 3.2)	< 0.001	26.0
History of Antibiotics use vs. No History of Antibiotics use	**13** [27, 30, 33, 38, 42, 44, 53, 57, 58, 61, 63, 73, 74]	1.4 (1.2–1.7)	0.001	8.5
Diabetes Mellitus vs. No Diabetes Mellitus	**2** [30, 63]	4.4 (1.3 − 14.6)	0.015	71.9
CD4 Count < 200 cells/µL vs. >500 cells/µL	**2** [40, 61]	2.8 (1.4–5.5)	0.002	0.0
Invasive procedures vs. No Invasive procedures	**2** [55, 61]	4.8 (1.9–12.6)	0.001	0.0
Nurse vs. Physician	**13** [22, 24, 26, 27, 31–33, 38, 54, 66, 68, 74, 82]	1.8 (1.3–2.5)	0.005	31.1
**b) Non-significant factors**				
Female vs. Male	**21** [22, 31, 33, 34, 40, 43, 44, 49, 53–56, 58, 61, 63, 65, 66, 73, 74, 78, 82]	1.2 (0.97–1.4)	0.11	74.5
Nasal allergy vs. No Nasal allergy	**3** [30, 34, 62]	2.0 (0.8–5.4)	0.15	49.3
Smoking vs. No Smoking	**4** [27, 30, 34, 55]	0.9 (0.6–1.3)	0.6	0.0

## Discussion

The meta-analysis, encompassing 23,484 participants from 69 studies, demonstrates significant variability in MRSA colonization across populations in Africa. Subgroup analyses revealed notably higher colonization rates among healthcare workers (13.6%) and hospitalized patients (12.9%) compared to healthy community residents (4.1%) and children (4.7%). Antibiotic resistance was low for linezolid (2.7%) and vancomycin (5.9%), but higher for mupirocin (10.7%), clindamycin (23.6%), and TMP-SMX (38.9%), highlighting the need for ongoing surveillance and targeted antibiotic stewardship. Key risk factors significantly associated with MRSA colonization (*p* < 0.05) include hospitalization (RR: 2.2), prior antibiotic use (RR: 1.4), diabetes mellitus (RR: 4.4), HIV with CD4 counts below 200 (RR: 2.8), invasive procedures (RR: 4.8), and being a nurse compared to a physician (RR: 1.8). These findings highlight the urgent need for targeted interventions to reduce MRSA colonization, particularly among high-risk populations who would benefit most from such measures.

Our findings revealed significant variations in colonization rates across different population groups. Specifically, our findings indicate that 4.1% of healthy community residents in the studied regions are MRSA carriers, a prevalence markedly higher than the estimate provided by the CDC, which approximates MRSA carriage in only 2% of the general population [91]. Regarding pediatric populations, a meta-analysis estimated the pooled prevalence of MRSA colonization among children to be 5% [92], a figure consistent with our estimate of 4.7% among African children. Furthermore, this study demonstrates that the prevalence of MRSA carriage among HCWs in Africa (13.6%) substantially exceeds rates reported in Europe and the United States, where a meta-analysis documented a pooled prevalence of 1.8% [93]. Similarly, a meta-analysis conducted in South Asia reported an overall prevalence of 9.23% among HCWs. However, significant heterogeneity was observed across countries, with prevalence rates ranging from 5.6% in India to 22.5% in Sri Lanka [94]. These disparities underscore the need for further research to elucidate the factors contributing to the varying burden of MRSA colonization across different regions and populations. The elevated prevalence of MRSA colonization among HCWs in Africa, with rates as high as 13.7% continent-wide and 18.1% in Egypt is of particular concern. Genetic typing in outbreak investigations has confirmed that HCWs play a key role in MRSA transmission [95]. A systematic review of MRSA outbreaks found that in 93% of studies using genotyping, HCWs were identified as the primary source of transmission to patients [95]. This highlights the urgent need for comprehensive mass decolonization programs and enhanced infection control strategies. These initiatives should be tailored to address the specific challenges in resource-limited settings, ensuring feasibility, sustainability, and compliance. Such measures are essential to effectively reduce the transmission of MRSA and alleviate the impact of healthcare-associated infections in these region.

Our findings revealed that the antibiotic resistance rates among MRSA isolates were relatively low for linezolid (2.7%) and vancomycin (5.9%). This aligns with previous meta-analyses from Egypt, which reported vancomycin and linezolid resistance rates of 9% and 5%, respectively, among clinically isolated MRSA [96]. Another meta-analysis from Ethiopia reported a pooled vancomycin resistance rate of 5.3% of isolates [97], while Tanzania reported an extremely high linezolid resistance rate of 20% among *S. aureus* isolates [98]. Although these resistance rates are relatively low, they exceed global averages and point to a concerning trend. Resistance to linezolid in MRSA is exceptionally rare. For instance, only 0.4% of MRSA isolates showed resistance to linezolid, and just 0.1% were resistant to vancomycin, according to the CDC’s National Healthcare Safety Network in 2021 [99]. In Europe, the rate of linezolid resistance among MRSA blood isolates was notably low at 0.29% [100]. Similarly, in China, no resistance to vancomycin or linezolid was detected among 9,116 MRSA isolates screened [101]. This is especially critical because linezolid and vancomycin are primary antibiotics used to treat severe MRSA infections. This high resistance to linezolid and vancomycin may be linked to widespread inappropriate use in Africa. For instance, a report from Egypt highlighted that linezolid was one of the major antibiotics dispensed for COVID-19 patients, with 13% of prescriptions occurring without proper clinical indication [102]. Similarly, in Tunisia, inappropriate use in ICU settings was reported, often without microbiological confirmation or consultation with infectious disease specialists [103].

Rifampin is a key antibiotic in combination therapy for MRSA, particularly in implant-associated and deep-seated infections [104]. It effectively penetrates biofilms and intracellular spaces [105]. However, it should never be used as monotherapy due to the high risk of resistance development [104, 106]. Resistance arises from single-step (point) mutations in the *rpoB* gene, which alter RNA polymerase [104, 106]. The high rifampin resistance rate among MRSA carriers in this study (62.7%) is likely driven by poor antibiotic stewardship, frequent monotherapy, and overuse in tuberculosis treatment, which is prevalent in African settings. We also revealed a pooled MRSA resistance rate of 10.7% for mupirocin. This finding is consistent with a systematic review and meta-analysis that reported a pooled prevalence of mupirocin-resistant MRSA in Iran at 9.9% [5.1–14.6] [10]. However, our results were higher than those observed in India 6.6% [1.8–11.5] and Korea 4.5% [1.6–7.4] [10]. The findings highlight the importance of continuous monitoring of mupirocin resistance in MRSA. Resistance can undermine its effectiveness as a decolonizing agent and elevate the risk of MRSA transmission within healthcare environments.

In terms of significant risk factors, this study identified hospitalization (RR: 2.2), prior antibiotic use (RR: 1.4), and invasive procedures (RR: 4.8) as significant contributors to MRSA colonization in Africa, aligning with findings from similar meta-analyses [92, 107]. Prior antibiotic use increases the risk of MRSA colonization by disrupting the normal bacterial flora, reducing competition, and allowing MRSA to thrive. Additionally, antibiotics exert selective pressure, favoring the survival of resistant strains like MRSA. The study also revealed that being a nurse, compared to a physician, is associated with an increased risk (RR: 1.8). A meta-analysis conducted in Europe and the United States found that nursing staff had the highest MRSA colonization rate at 6.9%, with an odds ratio of 1.72 (95% CI, 1.07–2.77) compared to medical staff [93]. Whether this increased risk is due to greater exposure and longer work hours for nurses or a potential lack of standardized hygiene knowledge compared to physicians requires further investigation. Similarly, immunocompromising conditions such as HIV and diabetes increase the risk, as indicated by RR values of 2.8 for HIV with CD4 counts less than 200 and 4.4 for diabetes mellitus, respectively. These findings align with previous meta-analyses [107, 108]. This increased risk is attributed to immunosuppression, which directly weakens immune defenses and indirectly increases exposure to MRSA through more frequent healthcare exposure. Regarding non-significant risk factors for MRSA colonization, this study found that gender was not a contributing factor. Previous findings on this topic have been inconsistent, with one meta-analysis suggesting males [107] and another suggesting females as being at higher risk [92]. These contradictions may indicate that gender alone is not a determinant of MRSA colonization, but rather other underlying factors, such as exposure, comorbidities, or healthcare-related risks, play a more significant role. In addition, nasal allergy was not identified as a definitive risk factor for MRSA colonization in this study. However, there is a noticeable trend, with a RR of 2.0 (95% CI: 0.8–5.4). Since this finding is based on only three studies, further investigation is required to determine whether nasal allergy is a significant risk factor.

### Strengths and limitations

This analysis demonstrates several notable strengths. It offers a broad overview of MRSA colonization in Africa without being restricted to specific subgroups, ensuring a comprehensive understanding of the issue. The rigorous methodology provides a detailed evaluation of MRSA colonization rates, antibiogram data, and associated risk factors across the continent. The inclusion of 69 studies, encompassing a substantial sample size of 23,484 participants, enhances the study’s reliability. Furthermore, the fair quality of the included studies supports the robustness and consistency of the findings. The stability of the pooled risk ratios is further confirmed through sensitivity analyses.

However, several limitations must be acknowledged. First, the generalizability of the findings is restricted, as data were available from only 16 countries. Second, while this meta-analysis was not pre-registered, we ensured strict adherence to the PRISMA guidelines. Third, stratification by country was not feasible for most subgroups due to the limited number of studies available for each category. Fourth, the high heterogeneity of the pooled prevalence data, as indicated by the high I-squared value, should be acknowledged. While heterogeneity is a common feature of meta-analyses of proportions [109], this variability may be attributed to country-specific variations. Fifth, the use of disc diffusion for vancomycin in MRSA is not recommended [110] and the low number of included studies (*n* = 5) may have influenced the pooled resistance findings. In light of these limitations, there is a clear need for additional research, particularly in underrepresented regions and countries with no available data. Addressing these gaps is essential to improve the understanding of MRSA carriage and to generate more comprehensive and representative estimates across Africa.

To reduce MRSA carriage in Africa, a multifaceted approach is essential. Screening and decolonization efforts should prioritize HCWs and hospitalized patients, particularly in high-prevalence or outbreak settings. Antibiotic stewardship and resistance surveillance are crucial for ensuring judicious antibiotic use. Strict infection control measures—including hand hygiene, contact precautions, and isolation—are vital in limiting cross-transmission. Strengthening laboratory capacity, workforce training, and implementing Whole Genome Sequencing enhance surveillance and outbreak control by identifying transmission chains and emerging resistance patterns, enabling more targeted interventions.

## Conclusion

MRSA carriage rates are particularly high among HCWs and hospitalized patients. Targeted interventions should focus on high-risk groups, including those with significant exposure risks, such as HCWs, hospital janitors, and hospitalized patients, as well as individuals with immunocompromising conditions, such as diabetes or AIDS. Strengthening infection control programs in hospitals and regulating antibiotic use in communities are critical measures to reduce MRSA colonization rates, thereby lowering transmission and the overall infection burden. Furthermore, ongoing surveillance is essential, especially given MRSA’s high resistance to mupirocin, a key antibiotic used in decolonization efforts.

## Electronic supplementary material

Below is the link to the electronic supplementary material.

Supplementary Material 1

## Data Availability

All data generated and analyzed throughout this study were included either in this article or its supplementary information file.

## Abbreviations

MRSA: Methicillin-Resistant Staphylococcus aureus
TMP-SMX: Trimethoprim-Sulfamethoxazole
ASPs: Antibiotic Stewardship Programs
CDC: Centers for Disease Control and Prevention
RR: Risk Ratio
Healthcare Workers: HCWs
AIDS: Acquired Immunodeficiency Syndrome
HIV: Human Immunodeficiency Virus
