# Supplementary material for: Prevalence, antibiogram, and risk factors of methicillin-resistant Staphylococcus aureus (MRSA) asymptomatic carriage in Africa: a systematic review and meta-analysis
Source: BMC Infect Dis. 2025 Apr 11;25:505. doi: 10.1186/s12879-025-10819-4 (PMC11987463; doi:10.1186/s12879-025-10819-4)
Supplement: Supplementary file 1 — Supplementary Material 1 [file 12879_2025_10819_MOESM1_ESM.docx]

**Additional file**

**Prevalence, Antibiogram, and Risk Factors of Methicillin-Resistant *Staphylococcus aureus* (MRSA) Asymptomatic Carriage in Africa: A Systematic Review and Meta-Analysis**

**Table S1**: The detailed search strategy

| **Category** | **Search Terms** |
| --- | --- |
| **MRSA Terms** | "Methicillin-resistant Staphylococcus aureus", "Methicillin resistant Staphylococcus aureus", MRSA, “Methicillin-resistant *S. aureus”* |
| **Colonization-Related Terms** | Carrier, Coloniz*, Colonis*, Carriage |
| **African Countries** | Africa*, Algeria, Angola, Benin, Botswana, "Burkina Faso", Burundi, "Cabo Verde", Cameroon, "Central African Republic", Chad, Comoros, Congo, "Democratic Republic of the Congo", Djibouti, Egypt, "Equatorial Guinea", Eritrea, Eswatini, Ethiopia, Gabon, Gambia, Ghana, Guinea, "Guinea-Bissau", "Ivory Coast", Kenya, Lesotho, Liberia, Libya, Madagascar, Malawi, Mali, Mauritania, Mauritius, Morocco, Mozambique, Namibia, Niger, Nigeria, Rwanda, "São Tomé and Príncipe", Senegal, Seychelles, "Sierra Leone", Somalia, "South Africa", "South Sudan", Sudan, Tanzania, Togo, Tunisia, Uganda, Zambia, Zimbabwe |

**Table S2**: The Detailed search strategy for Scopus and PubMed Databases

| Database | Search Strategy |
| --- | --- |
| Scopus | (TITLE-ABS-KEY ("Methicillin-resistant Staphylococcus aureus" OR "Methicillin resistant Staphylococcus aureus" OR MRSA OR "Methicillin-resistant S. aureus"))  AND (TITLE-ABS-KEY (Carrier OR Coloniz* OR Colonis* OR Carriage))  AND (TITLE-ABS-KEY (Africa* OR Algeria OR Angola OR Benin OR Botswana OR "Burkina Faso" OR Burundi OR "Cabo Verde" OR Cameroon OR "Central African Republic" OR Chad OR Comoros OR Congo OR "Democratic Republic of the Congo" OR Djibouti OR Egypt OR "Equatorial Guinea" OR Eritrea OR Eswatini OR Ethiopia OR Gabon OR Gambia OR Ghana OR Guinea OR "Guinea-Bissau" OR "Ivory Coast" OR Kenya OR Lesotho OR Liberia OR Libya OR Madagascar OR Malawi OR Mali OR Mauritania OR Mauritius OR Morocco OR Mozambique OR Namibia OR Niger OR Nigeria OR Rwanda OR "São Tomé and Príncipe" OR Senegal OR Seychelles OR "Sierra Leone" OR Somalia OR "South Africa" OR "South Sudan" OR Sudan OR Tanzania OR Togo OR Tunisia OR Uganda OR Zambia OR Zimbabwe))  AND (PUBYEAR >= 2014 AND PUBYEAR <= 2025) |
| PubMed | (("Methicillin-resistant Staphylococcus aureus"[Title/Abstract] OR "Methicillin resistant Staphylococcus aureus"[Title/Abstract] OR MRSA[Title/Abstract] OR "Methicillin-resistant S. aureus"[Title/Abstract])  AND (Carrier[Title/Abstract] OR Coloniz*[Title/Abstract] OR Colonis*[Title/Abstract] OR Carriage[Title/Abstract])  AND (Africa* OR Algeria OR Angola OR Benin OR Botswana OR "Burkina Faso" OR Burundi OR "Cabo Verde" OR Cameroon OR "Central African Republic" OR Chad OR Comoros OR Congo OR "Democratic Republic of the Congo" OR Djibouti OR Egypt OR "Equatorial Guinea" OR Eritrea OR Eswatini OR Ethiopia OR Gabon OR Gambia OR Ghana OR Guinea OR "Guinea-Bissau" OR "Ivory Coast" OR Kenya OR Lesotho OR Liberia OR Libya OR Madagascar OR Malawi OR Mali OR Mauritania OR Mauritius OR Morocco OR Mozambique OR Namibia OR Niger OR Nigeria OR Rwanda OR "São Tomé and Príncipe" OR Senegal OR Seychelles OR "Sierra Leone" OR Somalia OR "South Africa" OR "South Sudan" OR Sudan OR Tanzania OR Togo OR Tunisia OR Uganda OR Zambia OR Zimbabwe))  AND ("2014/01/01"[Date - Publication] : "2025/01/01"[Date - Publication]) |

**Table S3: The 27-Item Checklist of the PRISMA Statement**

| Section/topic | Item No | Checklist item | Reported on page No |
| --- | --- | --- | --- |
| Title | | | |
| Title | 1 | Identify the report as a systematic review, meta-analysis, or both | 1 |
| Abstract | | | |
| Structured summary | 2 | Provide a structured summary including, as applicable, background, objectives, data sources, study eligibility criteria, participants, interventions, study appraisal and synthesis methods, results, limitations, conclusions and implications of key findings, systematic review registration number | 2,3 |
| Introduction | | | |
| Rationale | 3 | Describe the rationale for the review in the context of what is already known | 5 |
| Objectives | 4 | Provide an explicit statement of questions being addressed with reference to participants, interventions, comparisons, outcomes, and study design (PICOS) | 5 |
| Methods | | | |
| Protocol and registration | 5 | Indicate if a review protocol exists, if and where it can be accessed (such as web address), and, if available, provide registration information including registration number | 17 |
| Eligibility criteria | 6 | Specify study characteristics (such as PICOS, length of follow-up) and report characteristics (such as years considered, language, publication status) used as criteria for eligibility, giving rationale | 6 |
| Information sources | 7 | Describe all information sources (such as databases with dates of coverage, contact with study authors to identify additional studies) in the search and date last searched | 5 |
| Search | 8 | Present full electronic search strategy for at least one database, including any limits used, such that it could be repeated | Tables S1 and S2 |
| Study selection | 9 | State the process for selecting studies (that is, screening, eligibility, included in systematic review, and, if applicable, included in the meta-analysis) | 6 |
| Data collection process | 10 | Describe method of data extraction from reports (such as piloted forms, independently, in duplicate) and any processes for obtaining and confirming data from investigators | 6&7 |
| Data items | 11 | List and define all variables for which data were sought (such as PICOS, funding sources) and any assumptions and simplifications made | 6&7 |
| Risk of bias in individual studies | 12 | Describe methods used for assessing risk of bias of individual studies (including specification of whether this was done at the study or outcome level), and how this information is to be used in any data synthesis | 7  **Table S4 and S5.** |
| Summary measures | 13 | State the principal summary measures (such as risk ratio, difference in means). | 7&8 |
| Synthesis of results | 14 | Describe the methods of handling data and combining results of studies, if done, including measures of consistency (such as I^2^ statistic) for each meta-analysis | 7&8 |
| Risk of bias across studies | 15 | Specify any assessment of risk of bias that may affect the cumulative evidence (such as publication bias, selective reporting within studies) | Not applicable |
| Additional analyses | 16 | Describe methods of additional analyses (such as sensitivity or subgroup analyses, meta-regression), if done, indicating which were pre-specified | 6&7 |
| Results | | | |
| Study selection | 17 | Give numbers of studies screened, assessed for eligibility, and included in the review, with reasons for exclusions at each stage, ideally with a flow diagram | 8&  Fig. 1 |
| Study characteristics | 18 | For each study, present characteristics for which data were extracted (such as study size, PICOS, follow-up period) and provide the citations | 9 and  Table S5. |
| Risk of bias within studies | 19 | Present data on risk of bias of each study and, if available, any outcome-level assessment (see item 12). | Table S4 and S5 |
| Results of individual studies | 20 | For all outcomes considered (benefits or harms), present for each study (a) simple summary data for each intervention group and (b) effect estimates and confidence intervals, ideally with a forest plot | Tables 1-3 and Figs 4-6 |
| Synthesis of results | 21 | Present results of each meta-analysis done, including confidence intervals and measures of consistency | Tables 1-3 and Figs 2&3 |
| Risk of bias across studies | 22 | Present results of any assessment of risk of bias across studies (see item 15) | Not applicable |
| Additional analysis | 23 | Give results of additional analyses, if done (such as sensitivity or subgroup analyses, meta-regression) (see item 16) | 9 &Table 1 |
| Discussion | | | |
| Summary of evidence | 24 | Summarize the main findings including the strength of evidence for each main outcome; consider their relevance to key groups (such as health care providers, users, and policy makers) | 14 |
| Limitations | 25 | Discuss limitations at study and outcome level (such as risk of bias), and at review level (such as incomplete retrieval of identified research, reporting bias) | 17&18 |
| Conclusions | 26 | Provide a general interpretation of the results in the context of other evidence, and implications for future research | 18 |
| Funding | | | |
| Funding | 27 | Describe sources of funding for the systematic review and other support (such as supply of data) and role of funders for the systematic review | 19 |

**Table S4**: The checklist items for Joanna Briggs's critical appraisal tool for prevalence studies

|  | Yes | No | Unclear | Not applicable |
| --- | --- | --- | --- | --- |
| 1. Was the sample frame appropriate to address the target population? | □ | □ | □ | □ |
| 1. Were study participants sampled in an appropriate way? | □ | □ | □ | □ |
| 1. Was the sample size adequate? | □ | □ | □ | □ |
| 1. Were the study subjects and the setting described in detail? | □ | □ | □ | □ |
| 1. Was the data analysis conducted with sufficient coverage of the identified sample? | □ | □ | □ | □ |
| 1. Were valid methods used for the identification of the condition? | □ | □ | □ | □ |
| 1. Was the condition measured in a standard, reliable way for all participants? | □ | □ | □ | □ |
| 1. Was there appropriate statistical analysis? | □ | □ | □ | □ |
| 1. Was the response rate adequate, and if not, was the low response rate managed appropriately? | □ | □ | □ | □ |

**Table S5**: characteristics of the included articles

| **Last Name of First Author** | **Publication Year** | **Country** | **Study Period** | **Study Population** | **Age of Participants (Years)** | **No. of Screened Participants** | **No. of MRSA Isolates (Prevalence %)** | **Specimen** | **Quality score (out of 9)** |
| --- | --- | --- | --- | --- | --- | --- | --- | --- | --- |
| **Abaza** [1] | **2016** | **Egypt** | **2012–2013** | **Community cases, HCWs, Patients** | **21–40** | **1021** | **81**  **(7.9%)** | **Nasal** | **8** |
| **Ali** [2] | **2019** | **Egypt** | **2016–2017** | **AD Patients** | **1–70** | **60** | **28**  **(46.6%)** | **Nasal, skin from AD lesions** | **7** |
| **Allam**[3] | **2021** | **Egypt** | **2018–2019** | **HCWs** | **18–57** | **163** | **45**  **(27.6%)** | **Nasal** | **8** |
| **Elzorkany** [4] | **2019** | **Egypt** | **2017** | **HD patients and HCWs** | **Mean: 49.18 years** | **285** | **54**  **(18.9%)** | **Nasal and hand** | **8** |
| **Fouda** [5] | **2016** | **Egypt** | **2013** | **ICU Patients** | **Mean: 47.75 (18–84)** | **92** | **58**  **(63%)** | **Throat, axillary, groin** | **7** |
| **Hamdy** [6] | **2024** | **Egypt** | **2019** | **Pharmacy students** | **19–23** | **196** | **12**  **(6.1%)** | **Nasal** | **8** |
| **Kady** [7] | **2015** | **Egypt** | **2015** | **Paramedical students** | **18–21** | **100** | **16**  **(16%)** | **Nasal** | **6** |
| **Awad** [8] | **2022** | **Egypt** | **2020–2021** | **HCWs, pediatric patients in NICU, PICU, and wards** | **HCWs: 18–54; Patients: <18** | **193** | **46**  **(23.8%)** | **Nasal and hand** | **7** |
| **Lashin** [9] | **2023** | **Egypt** | **2017** | **Inpatients, HCWs** | **>41** | **120** | **14**  **(11.7%)** | **Nasal and hand** | **7** |
| **Malek** [10] | **2019** | **Egypt** | **2018** | **HCWs** | **NA** | **150** | **22**  **(14.7%)** | **Nasal** | **8** |
| **Mostafa** [11] | **2022** | **Egypt** | **2019–2020** | **HCWs** | **NA** | **100** | **25**  **(25.0%)** | **Nasal** | **7** |
| **Salem** [12] | **2015** | **Egypt** | **2014** | **HCWs** | **Mean: 35.6** | **60** | **22**  **(36.67%)** | **Nasal and hand** | **6** |
| **Shady** [13] | **2015** | **Egypt** | **2011** | **Outpatients with no known risk factors for MRSA colonization** | **0.560** | **103** | **33**  **(32%)** | **Nasal** | **6** |
| **Taha** [14] | **2020** | **Egypt** | **2019** | **HCWs** | **<30 –≥45** | **223** | **22**  **(9.9%)** | **Nasal** | **7** |
| **Hefzy** [15] | **2016** | **Egypt** | **NA** | **HCWs** | **17–55** | **223** | **30**  **(13.5%)** | **Nasal** | **7** |
| **Al-Abdli** [16] | **2014** | **Libya** | **April 2013 – August 2013** | **HCWs** | **20–65** | **472** | **101**  **(21.4%)** | **Nasal** | **8** |
| **Alatery** [17] | **2016** | **Libya** | **2012** | **Renal transplant patients** | **16–67** | **60** | **22**  **(36.7%)** | **Nasal** | **6** |
| **Al-Haddad** [18] | **2017** | **Libya** | **2009 –2013** | **Inpatient and outpatient children, mothers of inpatient children, and HCWs** | **Children (0–15) and adults** | **758** | **70**  **(9.2%)** | **Nasal** | **7** |
| **Doro** [19] | **2016** | **Libya** | **2013** | **HCWs** | **Adults** | **408** | **14**  **(3.4%)** | **Nasal** | **8** |
| **Khnfar** [20] | **2019** | **Libya** | **2013** | **HCWs** | **Adults** | **102** | **22**  **(21.6%)** | **Nasal and hand** | **7** |
| **Krima** [21] | **2020** | **Libya** | **2019** | **ICU patients** | **18–75** | **250** | **45**  **(18%)** | **Nasal and throat** | **8** |
| **Moman** [22] | **2023** | **Libya** | **2017** | **HCWs** | **All ages (mean=34)** | **210** | **4**  **(1.9%)** | **Skin** | **8** |
| **Mahmoud** [23] | **2015** | **Sudan** | **2009 –2010** | **HCWs and Community individual** | **Adults** | **114** | **23**  **(20.2%)** | **Nasal** | **6** |
| **Ed-dyb** [24] | **2020** | **Morocco** | **2017 –2018** | **Outpatient Children** | **0.83 – 15** | **300** | **2**  **(0.7%)** | **Nasal** | **7** |
| **Oumokhtar** [25] | **2013** | **Morocco** | **2010** | **HD patients** | **Mean= 46.8** | **70** | **1**  **(1.4%)** | **Nasal** | **6** |
| **Antri** [26] | **2018** | **Algeria** | **2009 –2011** | **Healthy individual** | **0–79** | **459** | **24**  **(5.2%)** | **Nasal, Throat, Anal** | **8** |
| **Ouidri** [27] | **2018** | **Algeria** | **NA** | **Inpatients** | **0– >60** | **663** | **36**  **(5.4%)** | **Nasal** | **8** |
| **Job** [28] | **2018** | **Nigeria** | **2016** | **HIV-positive patients** | **>18** | **214** | **35**  **(16.4%)** | **Nasal** | **7** |
| **Adesida** [29] | **2016** | **Nigeria** | **2013** | **Hospitalized older adults** | **65–94** | **230** | **10**  **(4.3%)** | **Nasal** | **7** |
| **Ayepola** [30] | **2015** | **Nigeria** | **2010 – 2011** | **University Students and HCWS** | **Adults** | **73** | **2**  **(2.7%)** | **Nasal** | **6** |
| **Ifediora** [31] | **2023** | **Nigeria** | **NA** | **University students attending poultry farms** | **Adults** | **150** | **31**  **(20.7%)** | **Nasal** | **6** |
| **Jalmet** [32] | **2023** | **Nigeria** | **2016 –2017** | **School students** | **10–21** | **385** | **18**  **(4.7%)** | **Nasal** | **8** |
| **Nwokah** [33] | **2017** | **Nigeria** | **2014** | **HCWs** | **20–50** | **222** | **5**  **(2.3%)** | **Nasal and hand** | **8** |
| **Okwu** [34] | **2023** | **Nigeria** | **2019** | **Healthy students** | **16–33** | **100** | **6**  **(6%)** | **Nasal** | **7** |
| **Chukwuka** [35] | **2024** | **Nigeria** | **2022** | **University students** | **18–80** | **100** | **22**  **(22%)** | **Nasal** | **7** |
| **Onanuga (a)** [36] | **2019** | **Nigeria** | **2016** | **University students** | **15–35** | **400** | **31**  **(7.8%)** | **Nasal** | **8** |
| **Onanuga (b)** [37] | **2021** | **Nigeria** | **2018** | **Healthy adults** | **15–62** | **262** | **12**  **(4.6%)** | **Nasal** | **8** |
| **Oyekale**[38] | **2021** | **Nigeria** | **2020** | **HCWs** | **Mean= 32.9** | **135** | **36**  **(26.7%)** | **Nasal and hand** | **7** |
| **Wichendu** [39] | **2018** | **Nigeria** | **2018** | **HCWs** | **20–50 years** | **109** | **9**  **(8.3%)** | **Nasal** | **7** |
| **Ogefere** [40] | **2019** | **Nigeria** | **NA** | **University students** | **NA** | **350** | **55**  **(15.7%)** | **Nasal** | **8** |
| **Tuta** [41] | **2017** | **Nigeria** | **NA** | **Children** | **0.5–16** | **300** | **16**  **(5.3%)** | **Nasal** | **7** |
| **Anie** [42] | **2022** | **Nigeria** | **NA** | **University students** | **NA** | **300** | **68**  **(22.7%)** | **Nasal** | **8** |
| **Ajani** [43] | **2020** | **Nigeria** | **2018 - 2019** | **Medical students** | **15–25** | **200** | **4**  **(2%)** | **Nasal** | **8** |
| **Eibach** [44] | **2017** | **Ghana** | **2014 –2015** | **Children** | **< 15** | **544** | **2**  **(0.4%)** | **Nasal** | **8** |
| **Hogan** [45] | **2017** | **Madagascar** | **NA** | **HCWs, Non-medical university students** | **Adults** | **1548** | **20**  **(1.3%)** | **Nasal** | **8** |
| **Pietrzak** [46] | **2020** | **South Africa** | **2016** | **Patients undergoing elective total hip or knee arthroplasty** | **> 18** | **119** | **0**  **(0%)** | **Nasal, groin, and axillary** | **7** |
| **Mapanguy** [47] | **2024** | **Republic of Congo** | **2022** | **Healthy adults** | **18–28** | **175** | **4**  **(2.3%)** | **Nasal and oropharyngeal** | **7** |
| **Bebell** [48] | **2017** | **Uganda** | **2015** | **inpatients, outpatients** | **Mean = 37** | **500** | **14**  **(2.8%)** | **Nasal** | **7** |
| **Abimana** [49] | **2019** | **Uganda** | **2016–2017** | **HCWs** | **NA** | **97** | **8**  **(8.3%)** | **Nasal** | **6** |
| **Joachim (a)** [50] | **2018** | **Tanzania** | **2016** | **HCWs** | **20–61** | **379** | **59**  **(15.6%)** | **Nasal** | **8** |
| **Joachim (b)** [51] | **2017** | **Tanzania** | **2015** | **Patients admitted at emergency department** | **Mean = 34** | **258** | **22**  **(8.5%)** | **Nasal** | **8** |
| **Moyo** [52] | **2014** | **Tanzania** | **2010** | **Healthy children** | **Mean= 27.8 months** | **285** | **12**  **(4.2%)** | **Nasal** | **7** |
| **Khasabuli** [53] | **2017** | **Kenya** | **2015–2016** | **University students** | **18–30** | **237** | **26**  **(11%)** | **Nasal** | **7** |
| **Ita** [54] | **2022** | **Kenya** | **2019 –2020** | **Community individuals, inpatients** | **Children: Age < 5, Adults: Age ≥ 18** | **3018** | **52**  **(1.7%)** | **Nasal, Fecal, Rectal** | **8** |
| **Abie** [55] | **2020** | **Ethiopia** | **2020** | **Hospital and non-hospital janitors** | **18–58** | **436** | **22**  **(5%)** | **Nasal** | **8** |
| **Tigabu** [56] | **2018** | **Ethiopia** | **2018** | **School children** | **6–15 years** | **622** | **14**  **(2.3%)** | **Nasal** | **8** |
| **Belayhun** [57] | **2023** | **Ethiopia** | **2021** | **School children** | **7–14** | **384** | **58**  **(15.1%)** | **Nasopharyngeal** | **8** |
| **Birhanu** [58] | **2024** | **Ethiopia** | **2023** | **Outpatient children** | **Median=4** | **424** | **19**  **(4.5%)** | **Nasopharyngeal** | **8** |
| **Gebremedhn** [59] | **2016** | **Ethiopia** | **2014–2015** | **HIV-positive patients** | **5–72** | **249** | **6**  **(2.4%)** | **Nasal and Throat** | **7** |
| **Kahsay** [60] | **2018** | **Ethiopia** | **2017** | **Hospital and non-hospital janitors** | **18–24** | **384** | **24**  **(6.3%)** | **Nasal** | **8** |
| **Legese** [61] | **2018** | **Ethiopia** | **2016** | **HCWs** | **20–59** | **242** | **14**  **(5.8%)** | **Nasal** | **8** |
| **Reta (a)** [62] | **2015** | **Ethiopia** | **2013** | **School children** | **6–12** | **300** | **17**  **(5.7%)** | **Nasal** | **8** |
| **Reta (b)** [63] | **2017** | **Ethiopia** | **2015** | **Pre-school children** | **1–6** | **400** | **0**  **(0%)** | **Nasal** | **8** |
| **Shume** [64] | **2024** | **Ethiopia** | **2022** | **Medical students** | **19–29** | **250** | **12**  **(4.8%)** | **Nasal** | **8** |
| **Mekuriya** [65] | **2022** | **Ethiopia** | **2020** | **Medical students** | **21–27** | **258** | **19**  **(7.4%)** | **Nasal** | **8** |
| **Desta** [66] | **2022** | **Ethiopia** | **2018 - 2019** | **HCWs, administrative staff** | **20–57** | **1056** | **29**  **(2.8%)** | **Nasal** | **8** |
| **Walter** [67] | **2022** | **Namibia** | **2018** | **School children** | **5–14** | **272** | **48**  **(17.7%)** | **Nasal** | **8** |
| **Conceição** [68] | **2015** | **Angola** | **2014– 2015** | **Outpatient Children** | **0.83–17** | **158** | **20**  **(12.7%)** | **Nasal** | **8** |
| **Reid** [69] | **2017** | **Botswana** | **2013** | **HIV-positive patients** | **21– 68** | **404** | **13**  **(3.2%)** | **Nasal** | **8** |
| **AD: Atopic Dermatitis, HCWs: Health Care Workers, HD: Hemodialysis, ICU: Intensive Care Unit, NICU: Neonatal Intensive Care Unit, HIV: Human Immunodeficiency Virus** | | | | | | | | | |

1. Abaza AF, Mohamed ON, El-Fiky FK, Ahmed KA. Nasal carriage of methicillin-resistant Staphylococcus aureus and the effect of tea extracts on isolates. J Egypt Public Health Assoc. 2016;91:135–43.

2. Ali HA, El-Mahdy RH, Gaballah MA. Community-acquired methicillin-resistant Staphylococcus aureus colonization in atopic dermatitis patients in Mansoura, Egypt. Biomed Dermatology 2019 31. 2019;3:1–5.

3. Allam AAE, Fakhr AE, Mahmoud ME, El-Korashi LA. Staphylococcus aureus nasal colonization among health care workers at an Egyptian tertiary care hospital. Microbes Infect Dis. 2021;2:108–18.

4. Elzorkany KMA, Elbrolosy AM, Salem EH, Elzorkany KMA, Elbrolosy AM, Salem EH. Methicillin-Resistant Staphylococcus aureus Carriage in Hemodialysis Vicinity: Prevalence and Decolonization Approach. Indian J Nephrol. 2019;29:282–7.

5. Fouda R, Soliman MS, ElAnany MG, Abadeer M, Soliman G. Prevalence and risk factors of MRSA, ESBL and MDR bacterial colonization upon admission to an Egyptian medical ICU. J Infect Dev Ctries. 2016;10:329–36.

6. Hamdy A, Marciniak T, Alseqely M, Ziebuhr W, Abouelmagd E, Abouelfetouh A. Phenotypic and genotypic characterization of commensal staphylococci isolated from young volunteers in Alexandria, Egypt. Sci Reports 2024 141. 2024;14:1–11.

7. El-Kady H. Nasal Carriage of Staphylococcus aureus among Paramedical Students in Alexandria and Evaluation of Dry Spot Staphytect Latex Kit as a Rapid Screening Method for Staphylococcus aureus. J High Inst Public Heal. 2015;45:52–61.

8. Awad SM, Abd Elaziz AM, Adwey NM, Abou Hind ES. Molecular characteristics and carriage rate of Methicillin- Resistant Staphylococcus aureus (MRSA) in pediatric hepatology unit. Egypt J Med Microbiol. 2022;31:33–43.

9. Lashin GMA, Tohamy E, Ahmed HA, Rofida ;, Bakry M. Molecular studies of Staphylococcus aureus carriage in healthcare workers, patients and inanimate object in Zagazig hospitals. Bull Fac Sci Zagazig Univ. 2023;2023:192–207.

10. Malek MM, Abo-Alella DA. Methicillin-Resistant Staphylococcus Aureus Nasal Carriage among Health Care Workers in Surgery Department at a Tertiary Care Hospital in Egypt. Egypt J Med Microbiol. 2019;28:115–20.

11. Mostafa G, Badr MFA, Zeid MS, Eldegla H. Nasal Carriage of Community Acquired and Inducible Dormant Methicillin Resistant Staphylococcus aureus among Healthcare Workers of Mansoura University Children’s Hospital. Egypt J Med Microbiol. 2022;31:75–81.

12. Salem EA, El-Masry E, El-Dalatony MM. Staphylococcus Aureus Carriage among Healthcare Workers in Burn and Surgical Critical Care Units in Menoufia University Hospital, Egypt. J High Inst Public Heal. 2015;45:8–15.

13. Abou Shady HM, Bakr AEA, Hashad ME, Alzohairy MA. Staphylococcus aureus nasal carriage among outpatients attending primary health care centers: a comparative study of two cities in Saudi Arabia and Egypt. Braz J Infect Dis. 2015;19:68–76.

14. Taha MS, Younis EA, Hegazy EE. Nasal Carriage Rate and Antimicrobial Resistance Profiles of Methicillin-Resistant Staphylococcus Aureus (MRSA) among Health Care Workers in Tanta University Hospital. Egypt J Med Microbiol. 2020;29:1–7.

15. Hefzy EM, Hassan GM, Abd El Reheem F. Detection of Panton-Valentine Leukocidin-Positive Methicillin-Resistant Staphylococcus aureus Nasal Carriage among Egyptian Health Care Workers. Surg Infect (Larchmt). 2016;17:369–75.

16. E. Al-Abdli N, Baiu SH. Nasal Carriage of Staphylococcus in Health Care Workers in Benghazi Hospitals. Am J Microbiol Res. 2014;2:110–2.

17. Alatery A, Besher F, Shubar H, Al-megrahi N. Prevalence and Clinical Significance of Nasal Colonization with Multi-drug Resistant Bacteria on Libyan Kidney Recipients‏. Tripolitana Med J. 2016;5:23–32.

18. Al-haddad OH, Zorgani A, Ghenghesh KS. Nasal carriage of multi-drug resistant Panton-Valentine leucocidin-positive methicillin-resistant Staphylococcus aureus in children in Tripoli-Libya. Am J Trop Med Hyg. 2014;90:724–7.

19. Doro B, Zawia W, Gafri F, Abogress O, Habishi M, Zawia A. Prevalence of Methicillin-resistant Staphylococcus aureus among Health Care Workers in Tripoli Hospital, Libya. Br Microbiol Res J. 2016;14:1–7.

20. Khnfar HYH, Thaood DA, Abdraba IOA, Omran IN, Abdeldaim GMK. Nasal and Hands Carriage Rate of Methicillin-Resistant Staphylococcus aure-us among Health Care Workers at Alwahda Hospital, Derna. Al-Mukhtar J Sci. 2019;34:279–87.

21. M. Krima H, El-Agheli SM, Sanger B. Prevalence of Methicillin Resistant Staphylococcus aureus isolates from Al-Khoms hospital medical staff, Libya. IOSR J Dent Med Sci. 2014;13:46–50.

22. Moman R, Moman RM, Alsaid AM, Furarah AM. Prevalence of nasal carriage of methicillin-resistant Staphylococcus aureus among healthcare workers in Tarhuna and Mesllata hospitals. Emerg Technol Innov Res. 2023;10.

23. Mahmoud AM, Albadawy HS, Bolis SM, Bilal NE, Ahmed AO, Ibrahim ME. Inducible clindamycin resistance and nasal carriage rates of Staphylococcus aureus among healthcare workers and community members. Afr Health Sci. 2015;15:861.

24. S E, M A, F A, W Q, N A, S A, et al. Prevalence of Community Acquired Methicillin Resistant Staphylococcus aureus Nasal Carriage among Children of Consultation: Experience of a Moroccan University Hospital. Arch Clin Microbiol. 2020;11.

25. Oumokhtar B, Elazhari M, Timinouni M, Bendahhou K, Bennani B, Mahmoud M, et al. Staphylococcus aureus nasal carriage in a Moroccan dialysis center and isolates characterization. Hemodial Int. 2013;17:542–7.

26. Antri K, Akkou M, Bouchiat C, Bes M, Martins-Simoes P, Dauwalder O, et al. High levels of Staphylococcus aureus and MRSA carriage in healthy population of Algiers revealed by additional enrichment and multisite screening. Eur J Clin Microbiol Infect Dis. 2018;37:1521–9.

27. Ouidri MA. Screening of nasal carriage of methicillin-resistant Staphylococcus aureus during admission of patients to Frantz Fanon Hospital, Blida, Algeria. New microbes new Infect. 2018;23:52–60.

28. Job P, Alo M, Nwokah E, Aaron U, 2018‏ U. Nasal Carriage Prevalence of MRSA in People Living with HIV/AIDS Undertaking Antiretroviral Therapy in a Tertiary Hospital in Port Harcourt‏. Int J Innov Healthc Res. 2018;6:12–23.

29. Adesida SA, Okeyide AO, Abioye A, Omolopo I, Egwuatu TO, Amisu KO, et al. Nasal Carriage of Methicillin-resistant Staphylococcus aureus among Elderly People in Lagos, Nigeria. Avicenna J Clin Microbiol Infect. 2016;3:39272–39272.

30. Ayepola OO, Olasupo NA, Egwari LO, Becker K, Schaumburg F. Molecular Characterization and Antimicrobial Susceptibility of Staphylococcus aureus Isolates from Clinical Infection and Asymptomatic Carriers in Southwest Nigeria. PLoS One. 2015;10:e0137531.

31. Ifediora A, Enya E. Variations In The Genotypic And Phenotypic Characteristics Of Methicillin-Resistant Staphylococcus Aureus (Mrsa) From Students Attending Poultry Farms In Umuahia, Abia State, Nigeria. Stamford J Microbiol. 2023;13:6–10.

32. Jalmet SY, Abimiku RH, Tama SC, Attah CJ, Okolo MO. Isolation and Molecular Detection of Mec-A Gene in Methicillin Resistant Staphylococcus aureus Colonizing Anterior Nares of School Children in Jos South, Nigeria. Asian J Biotechnol Genet Eng. 2023;6:87–95.

33. Nwokah E, Eddeh-Adjugah O, Aleru C, 2017‏ U. Assessment of asymptomatic methicillin resistant Staphylococcus aureus carriage among health care workers in the University of Port Harcourt teaching ‏. SCIREA J Heal. 2017;2:13–25.

34. Okwu MU, Akpoka AO, Mitsan O, Izevbuwa OE, Osamede A, Tkadlec J. High Frequency of Methicillin-Resistant and Multidrug-Resistant Strains of Staphylococcus aureus Colonizing Students in Okada, Edo State, Nigeria. Microb Drug Resist. 2023;29:516–22.

35. Enwa FO, Amaihunwa KC, Adjekuko CO, Onyolu SB. Prevalence of Community-Acquired Methicillin-Resistant Staphylococcus aureus (CA-MRSA) in the Nasal Carriage of Delta State University Students. J Biochem Technol. 2023;14:67–71.

36. Onanuga A, Eboh DD, Okou GT. Antibiogram and Virulence Characteristics of Multi-drug Resistant Staphylococcus aureus from Nasal Cavity of Healthy Students of Niger Delta University, Amassoma, Bayelsa State, Nigeria. J Clin Diagnostic Res. 2019;13:24–9.

37. Onanuga A, Adamu OJ, Odetoyin B, Hamza JA. Nasal Carriage of Multi-Drug Resistant Panton Valentine Leukocidin Positive Staphylococcus Aureus in Healthy Individuals of Tudun-Wada, Gombe State, Nigeria. African J Infect Dis. 2021;15:24–33.

38. Oyekale OT, Olajide AT, Ojo BO, Babalola TO, Oyekale OI. Carriage Rate of Methicillin-Resistant Staphylococcus aureus among Workers in Critical Care Units of a Tertiary Hospital in Southwestern Nigeria. J Adv Med Med Res. 2021;33:13–21.

39. Wichendu P, Wariso K, Igunma A, Olonipile F, Awopeju A, Dodiyi-Manuel A, et al. Nasal Carriage Rate Of Methicillin Resistant Staphylococcus Aureus Among Theatre Staff Of Upth, Rivers State, Nigeria. IOSR J Dent Med Sci. 2018;17:64–9.

40. Ogefere HO, Umaru G, Ibadin EE, Omoregie R. Prevalence Of Methicillin-Resistant Staphylococci Among Apparently Healthy Students Attending A Tertiary Institution In Benin City, Nigeria. Niger J Basic Appl Sci. 2019;27:114–21.

41. Tuta KE, Okesola AO, Umeokonkwo CD. The Prevalence and Risk Factors Associated with Nasal Methicillin-Resistant Staphylococcus Aureus Colonization among Children in a Tertiary Hospital in Nigeria. Ethiop J Health Sci. 2019;29:487–94.

42. Anie CO, Ibezim EC, Esimone CO, Arhewoh MI. Molecular characterization of selected nasal isolates of methicillin-resistant Staphylococcus aureus (MRSA) from healthy students of a tertiary institution. Trop J Pharm Res. 2022;21:825–31.

43. Ajani TA, Elikwu CJ, Nwadike V, Babatunde T, Anaedobe CG, Shonekan O, et al. Nasal carriage of methicillin resistant Staphylococcus aureus among medical students of a private institution in Ilishan-Remo, Ogun State, Nigeria. African J Clin Exp Microbiol. 2020;21:311–7.

44. Eibach D, Nagel M, Hogan B, Azuure C, Krumkamp R, Dekker D, et al. Nasal Carriage of Staphylococcus aureus among Children in the Ashanti Region of Ghana. PLoS One. 2017;12:e0170320.

45. Hogan B, Rakotozandrindrainy R, Al-Emran H, Dekker D, Hahn A, Jaeger A, et al. Prevalence of nasal colonisation by methicillin-sensitive and methicillin-resistant Staphylococcus aureus among healthcare workers and students in Madagascar. BMC Infect Dis. 2016;16:1–9.

46. Pietrzak JRT, Maharaj Z, Mokete L. Prevalence of Staphylococcus aureus colonization in patients for total joint arthroplasty in South Africa. J Orthop Surg Res. 2020;15:123.

47. Chastel C, Mapanguy M, Limingui JN, Schaumburg F, Elton L, Dareine Bitoumbou E, et al. Journal of Antibiotics and Antimicrobial Agents Characterization of Staphylococcus Aureus Carriage Among Asymptomatic Indi-viduals in the Republic of Congo. J Antibiot Antimicrob Agents. 2024;1.

48. Bebell LM, Ayebare A, Boum Y, Siedner MJ, Bazira J, Schiff SJ, et al. Prevalence and correlates of MRSA and MSSA nasal carriage at a Ugandan regional referral hospital. J Antimicrob Chemother. 2017;72:888–92.

49. Abimana JB, Kato CD, Bazira J. Methicillin-Resistant Staphylococcus aureus Nasal Colonization among Healthcare Workers at Kampala International University Teaching Hospital, Southwestern Uganda. Can J Infect Dis Med Microbiol. 2019;2019:4157869.

50. Joachim A, Moyo SJ, Nkinda L, Majigo M, Rugarabamu S, Mkashabani EG, et al. Nasal carriage of methicillin-resistant staphylococcus aureus among health care workers in tertiary and regional hospitals in Dar es Salam, Tanzania. Int J Microbiol. 2018;2018:5058390.

51. Joachim A, Moyo SJ, Nkinda L, Majigo M, Mbaga E, Mbembati N, et al. Prevalence of methicillin-resistant Staphylococcus aureus carriage on admission among patients attending regional hospitals in Dar es Salaam, Tanzania. BMC Res Notes. 2017;10:1–7.

52. Moyo SJ, Aboud S, Blomberg B, Mkopi N, Kasubi M, Manji K, et al. High nasal carriage of methicillin-resistant staphylococcus aureus among healthy tanzanian under-5 children. Microb Drug Resist. 2014;20:82–8.

53. O Y K, Ngugi C, Kiiru J. Carriage, antimicrobial susceptibility profiles and genetic diversity of Staphylococcus aureus and MRSA isolates recovered from students in a Kenyan university. East Afr Med J. 2017;94:506–23.

54. Ita T, Luvsansharav UO, Smith RM, Mugoh R, Ayodo C, Oduor B, et al. Prevalence of colonization with multidrug-resistant bacteria in communities and hospitals in Kenya. Sci Rep. 2022;12:1–9.

55. Abie S, Tiruneh M, Abebe W. Methicillin-resistant Staphylococcus aureus nasal carriage among janitors working in hospital and non-hospital areas: a comparative cross-sectional study. Ann Clin Microbiol Antimicrob. 2020;19:1–8.

56. Tigabu A, Tiruneh M, Mekonnen F. Nasal Carriage Rate, Antimicrobial Susceptibility Pattern, and Associated Factors of Staphylococcus aureus with Special Emphasis on MRSA among Urban and Rural Elementary School Children in Gondar, Northwest Ethiopia: A Comparative Cross-Sectional Study. Adv Prev Med. 2018;2018:9364757.

57. Belayhun C, Tilahun M, Seid A, Shibabaw A, Sharew B, Belete MA, et al. Asymptomatic nasopharyngeal bacterial carriage, multi-drug resistance pattern and associated factors among primary school children at Debre Berhan town, North Shewa, Ethiopia. Ann Clin Microbiol Antimicrob. 2023;22:1–16.

58. Birhanu A, Amare A, Tigabie M, Getaneh E, Assefa M, Cherkos T, et al. Nasopharyngeal carriage, antimicrobial susceptibility patterns, and associated factors of Gram-positive bacteria among children attending the outpatient department at the University of Gondar Comprehensive Specialized Hospital, Northwest Ethiopia. PLoS One. 2024;19:e0308017.

59. Gebremedhn G, Gebremariam TT, Wasihun AG, Dejene TA, Saravanan M. Prevalence and risk factors of methicillin-resistant Staphylococcus aureus colonization among HIV patients in Mekelle, Northern Ethiopia. Springerplus. 2016;5:1–9.

60. Kahsay AG, Hagos DG, Abay GK, Mezgebo TA. Prevalence and antimicrobial susceptibility patterns of methicillin-resistant Staphylococcus aureus among janitors of Mekelle University, North Ethiopia. BMC Res Notes. 2018;11:1–6.

61. Legese H, Kahsay AG, Kahsay A, Araya T, Adhanom G, Muthupandian S, et al. Nasal carriage, risk factors and antimicrobial susceptibility pattern of methicillin resistant Staphylococcus aureus among healthcare workers in Adigrat and Wukro hospitals, Tigray, Northern Ethiopia. BMC Res Notes. 2018;11:1–6.

62. Reta, A Gedefaw, L Sewunet T, Beyene G. Nasal Carriage, Risk Factors and Antimicrobial Susceptibility Pattern of Methicillin Resistant Staphylococcus aureus among School Children in Ethiopia. J Med Microbiol Diagnosis. 2015;04.

63. Reta A, Wubie M, Mekuria G. Nasal colonization and antimicrobial susceptibility pattern of Staphylococcus aureus among pre-school children in Ethiopia. BMC Res Notes. 2017;10:1–7.

64. Shume T, Urgesa K, Mekonnen S, Ayele F, Tesfa T, Tebeje F, et al. Nasal carriage of MRSA among clinically affiliated undergraduate students at the College of Health and Medical Sciences, Haramaya University, Ethiopia. Sci Rep. 2024;14:1–9.

65. Mekuriya E, Manilal A, Aklilu A, Woldemariam M, Hailu T, Wasihun B. Methicillin-resistant Staphylococcus aureus colonization among medicine and health science students, Arba Minch University, Ethiopia. Sci Rep. 2022;12:1–11.

66. Desta K, Aklillu E, Gebrehiwot Y, Enquselassie F, Cantillon D, Al-Hassan L, et al. High Levels of Methicillin-Resistant Staphylococcus aureus Carriage Among Healthcare Workers at a Teaching Hospital in Addis Ababa Ethiopia: First Evidence Using mecA Detection. Infect Drug Resist. 2022;15:3135–47.

67. Walter S, Beukes M, Mumbengegwi D, Böck R. Methicillin-resistant staphylococci among school children in Mariental, Namibia. Sci African. 2022;15:e01090.

68. Conceição T, Coelho C, Santos Silva I, De Lencastre H, Aires-De-Sousa M. Methicillin-Resistant Staphylococcus aureus in the Community in Luanda, Angola: Blurred Boundaries with the Hospital Setting. Microb Drug Resist. 2016;22:22–7.

69. Reid MJA, Steenhoff AP, Mannathoko N, Muthoga C, McHugh E, Brown EL, et al. Staphylococcus aureus nasal colonization among HIV-infected adults in Botswana: prevalence and risk factors. AIDS Care. 2017;29:961–5.
